# Phosphoproteomics Uncovers Exercise Intensity-Specific Skeletal Muscle Signaling Networks Underlying High-Intensity Interval Training in Healthy Male Participants

**DOI:** 10.1007/s40279-025-02217-2

**Published:** 2025-04-21

**Authors:** Nolan J. Hoffman, Jamie Whitfield, Di Xiao, Bridget E. Radford, Veronika Suni, Ronnie Blazev, Pengyi Yang, Benjamin L. Parker, John A. Hawley

**Affiliations:** 1 Exercise and Nutrition Research Program, Mary MacKillop Institute for Health Research, Australian Catholic University, Level 5, 215 Spring Street, Melbourne, VIC 3000 Australia; 2 Computational Systems Biology Unit, Children’s Medical Research Institute, Faculty of Medicine and Health, The University of Sydney, Westmead, NSW Australia; 3 School of Mathematics and Statistics and Charles Perkins Centre, The University of Sydney, Sydney, NSW Australia; 4 Department of Anatomy and Physiology, The University of Melbourne, Parkville, VIC Australia; 5 Centre for Muscle Research, The University of Melbourne, Parkville, VIC Australia; 6 Department of Sport and Exercise Sciences, Manchester Metropolitan University Institute of Sport, Manchester, UK

## Abstract

**Background:**

In response to exercise, protein kinases and signaling networks are engaged to blunt homeostatic threats generated by acute contraction-induced increases in skeletal muscle energy and oxygen demand, as well as serving roles in the adaptive response to chronic exercise training to blunt future disruptions to homeostasis. High-intensity interval training (HIIT) is a time-efficient exercise modality that induces superior or similar health-promoting skeletal muscle and whole-body adaptations compared with prolonged, moderate-intensity continuous training (MICT). However, the skeletal muscle signaling pathways underlying HIIT’s exercise intensity-specific adaptive responses are unknown.

**Objective:**

We mapped human muscle kinases, substrates, and signaling pathways activated/deactivated by an acute bout of HIIT versus work-matched MICT.

**Methods:**

In a randomized crossover trial design (Australian New Zealand Clinical Trials Registry number ACTRN12619000819123; prospectively registered 6 June 2019), ten healthy male participants (age 25.4 ± 3.2 years; BMI 23.5 ± 1.6 kg/m^2^; $\dot{V}{\text{O}}_{2} \max$ 37.9 ± 5.2 ml/kg/min, mean values ± SD) completed a single bout of HIIT and MICT cycling separated by ≥ 10 days and matched for total work (67.9 ± 10.2 kJ) and duration (10 min). Mass spectrometry-based phosphoproteomic analysis of muscle biopsy samples collected before, during (5 min), and immediately following (10 min) each exercise bout, to map acute temporal signaling responses to HIIT and MICT, identified and quantified 14,931 total phosphopeptides, corresponding to 8509 phosphorylation sites.

**Results:**

Bioinformatic analyses uncovered exercise intensity-specific signaling networks, including > 1000 differentially phosphorylated sites (± 1.5-fold change; adjusted *P* < 0.05; ≥ 3 participants) after 5 min and 10 min HIIT and/or MICT relative to rest. After 5 and 10 min, 92 and 348 sites were differentially phosphorylated by HIIT, respectively, versus MICT. Plasma lactate concentrations throughout HIIT were higher than MICT (*P* < 0.05), and correlation analyses identified > 3000 phosphosites significantly correlated with lactate (*q* < 0.05) including top functional phosphosites underlying metabolic regulation.

**Conclusions:**

Collectively, this first global map of the work-matched HIIT versus MICT signaling networks has revealed rapid exercise intensity-specific regulation of kinases, substrates, and pathways in human skeletal muscle that may contribute to HIIT’s skeletal muscle adaptations and health-promoting effects.

Preprint: The preprint version of this work is available on medRxiv, https://doi.org/10:1101/2024.07.11.24310302.

**Supplementary Information:**

The online version contains supplementary material available at 10.1007/s40279-025-02217-2.

## Key Points

**Table:** 

The breadth of common and unique signaling networks underlying human skeletal muscle adaptive responses to high-intensity interval training (HIIT) versus work-matched moderate-intensity continuous training (MICT) is unknown.
Global phosphoproteomic analysis of skeletal muscle biopsies from ten healthy male participants’ randomized crossover trials mapped rapid exercise signaling network responses to an acute bout of HIIT versus workload- and duration-matched MICT.
Pre-trial standardization achieved highly reproducible baseline signaling signatures between crossover trials.
More phosphosites were down- versus up-regulated in response to each exercise intensity and timepoint, suggesting acute exercise-regulated inhibition of kinase activity and/or activation of phosphatases.
Networks of exercise intensity-specific kinases, substrates, and pathways highly associated with plasma lactate concentrations were identified that may contribute to exercise’s health-promoting effects.

## Introduction

Exercise training confers numerous beneficial physiological adaptations in skeletal muscle, imparting a wide range of whole-body health benefits that can prevent, delay, and/or treat a range of chronic metabolic conditions including obesity, type 2 diabetes, and cardiovascular disease [1, 2]. Protein kinases and downstream signal transduction networks are activated/deactivated in contracting skeletal muscles in response to an acute bout of exercise in order to meet increased energy and oxygen demands and maintain cellular energy homeostasis. Over time (weeks to months), these repeated bouts of exercise lead to beneficial training adaptations [3, 4]. Protein phosphorylation is a key post-translational modification that regulates nearly all cellular biological processes, including skeletal muscle adaptations to exercise. Such adaptations are influenced by factors such as the mode, intensity, duration, and frequency of exercise by engaging distinct molecular transducers [5].

High-intensity interval training (HIIT) has attracted widespread scientific and popular interest as a low-volume, time-effective exercise intervention capable of inducing superior [6] or similar [7] physiological adaptations (e.g., increased cardiorespiratory fitness) and reductions in cardiometabolic disease risk factors compared with higher-volume, moderate-intensity continuous training (MICT). HIIT-based exercise protocols involve multiple (i.e., 4–10) work bouts of high-intensity exercise (≥ 80% $\dot{V}{\text{O}}_{2} \max$) interspersed with periods of rest or active recovery [8, 9]. A single bout of HIIT activates several key exercise-regulated kinases in skeletal muscle, including the AMP-activated protein kinase (AMPK) and p38 mitogen-activated protein kinase (p38MAPK), stimulating mitochondrial biogenesis and leading to increased mitochondrial content and enzyme activity in as little as 24 h post-exercise [10, 11]. Relative to a workload-matched bout of MICT, a single bout of HIIT elicits similar skeletal muscle exercise-induced increases in AMPK signaling [12], as well as p38MAPK and p53 tumor suppressor protein (p53) signaling responses that underpin mitochondrial biogenesis in human skeletal muscle [13]. In contrast, increased activation of key signaling pathways, including AMPK, p38 MAPK, and Ca^2+^/calmodulin-dependent protein kinase II (CAMKII), has also been detected in response to work-matched interval versus continuous exercise [14]. However, in-depth analyses of the signaling networks engaged by HIIT versus MICT are lacking, with studies to date having analyzed only a subset of key exercise-regulated kinases, without investigating the breadth of potential signaling pathways underlying each exercise intervention.

Global mass spectrometry (MS)-based phosphoproteomics has the capability of identifying and quantifying thousands of protein phosphorylation events occurring within the complex and interconnected signaling networks engaged in response to exercise [15]. The first global phosphoproteomic analysis of exercise signaling in human skeletal muscle uncovered > 1000 sites differentially phosphorylated after a short bout of continuous intense exercise (i.e., 8–12 min) versus rest [16]. Recently, Blazev et al. utilized phosphoproteomics to map human skeletal muscle canonical signaling network responses to an acute bout of endurance (90 min cycling at 60% $\dot{V}{\text{O}}_{2} \max$), sprint (cycling all-out for 3 × 30 s), and resistance exercise (six sets of ten repetition maximum knee extensions) in eight healthy male participants, revealing 420 core phosphosites common to all exercise modalities [17]. This study also identified divergent signaling responses to these different modalities of exercise, although the exercise bouts were not matched for total workload nor duration. As such, it is difficult to isolate the divergent signaling responses related to exercise intensity, as kinase regulation can display different time profiles of activation/deactivation [17]. Furthermore, the common and unique kinases and downstream signaling pathways engaged by work-matched HIIT versus MICT in human skeletal muscle remain unexplored with no global phosphoproteomic analyses of work-matched exercise performed to date.

Therefore, the overall aim of this study was to determine the temporal regulation of kinases and downstream signaling pathways by an acute bout of HIIT versus MICT within the same human participants while controlling for total exercise duration, workload, and diet. We hypothesized that HIIT would engage a unique subset of kinases and signaling pathways owing to the intense, intermittent nature of this exercise modality, including increased activation of signaling networks underlying mitochondrial biogenesis. Utilizing a phosphoproteomic approach, this is the first study to map the human skeletal muscle signaling networks underlying an acute bout of HIIT and across different work-matched exercise intensities. We identify > 1000 sites significantly regulated during (5 min) and immediately following (10 min) HIIT and/or MICT, including known and novel exercise-regulated signaling events. Furthermore, we identify a subset of kinases, substrates, and pathways differentially regulated by HIIT relative to MICT and highly associated with plasma lactate responses to exercise, revealing the molecular framework underlying adaptive responses to HIIT that become rapidly engaged and potentially contribute to HIIT’s muscle physiological adaptations and whole-body health benefits.

## Methods

### Human Participants

**Fig. 1:**
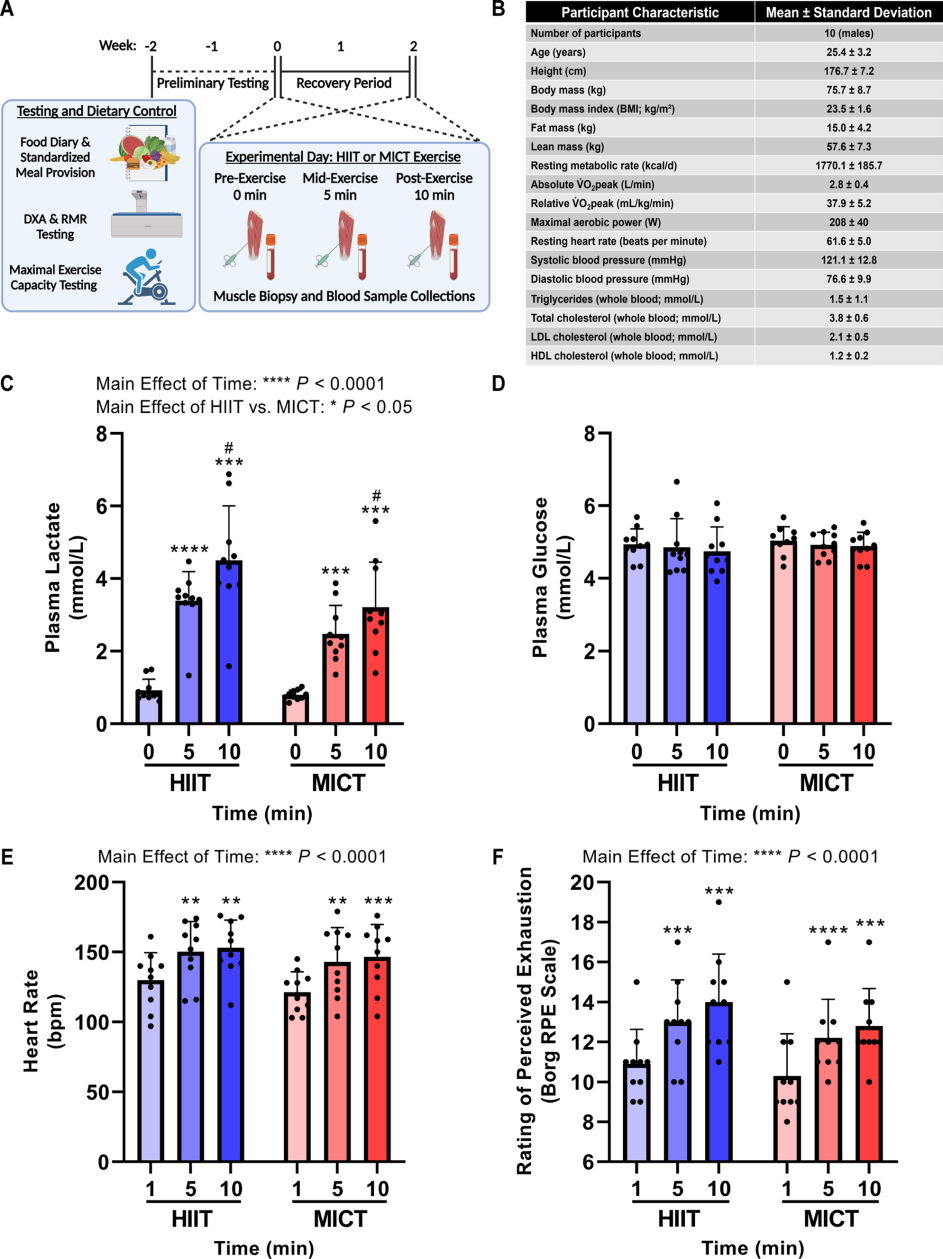
Preliminary testing and randomized crossover trial design, participant baseline characteristics, and plasma lactate and glucose responses to HIIT and MICT. As detailed in the overall study schematic (**A**), participants first underwent preliminary testing and dietary control prior to each experimental HIIT or MICT trial day. Participants arrived at the laboratory following overnight fasting for baseline measurements, a dual-energy X-ray absorptiometry (DXA) body composition scan and resting metabolic rate (RMR) testing. Each participant then completed an incremental fitness test to volitional fatigue on a cycle ergometer to determine peak oxygen uptake ($\dot{V}{\text{O}}_{{2}} {\text{peak}}$) and maximal aerobic power (MAP) to calculate the work rate for the subsequent two workload (67.9 ± 10.2 kJ) and total duration (10 min) matched HIIT and MICT exercise trials. Participants’ food and fluid intake for all meals and snacks was recorded over a 3-day period using a mobile phone application and analyzed by an accredited research dietician. A standardized dinner was consumed by each participant the evening prior to each exercise trial, with no caffeine or alcohol consumed 20 or 24 h prior, respectively. In a randomized crossover design, participants were randomly assigned their first exercise trial (i.e., HIIT or MICT) prior to commencing trial days and did not perform any exercise in the 72 h prior to each trial day. HIIT and MICT exercise trials were separated by at least a 10-day recovery period. On each experimental day, participants reported to the laboratory following overnight fasting, and vastus lateralis skeletal muscle biopsies and venous blood samples were collected pre-exercise (0 min), mid-exercise (5 min), and immediately post-exercise (10 min). Participant characteristics are listed in (**B**). Plasma (**C**) lactate and (**D**) glucose concentrations across the acute HIIT and MICT exercise bouts were determined using a YSI Analyzer. No interaction effect was observed for plasma lactate in (**C**); *P* = 0.0573. Heart rate (**E**) and rating of perceived exhaustion (**F**; RPE; Borg RPE scale out of 20) were recorded at 1 min (i.e., following completion of the first HIIT “on” interval), 5 min, and 10 min during HIIT or MICT trials. Data are presented as mean ± SD; two-way ANOVA with repeated measurements, Tukey’s test for multiple comparisons; **P < 0.01 versus 0 min (or 1 min in **E** and **F**); ***P < 0.001 versus 0 min (or 1 min in **E** and **F**); ****P < 0.0001 versus 0 min (or 1 min in **E** and **F**); ^#^*P* < 0.05 versus 5 min; *n* = 10 for each exercise intensity and timepoint

Ten healthy males aged 18–30 years with a body mass index 18.5–27.0 kg/m^2^ were recruited to participate in this study. Participant characteristics are shown in Fig. 1B. Inclusion criteria were physical inactivity (i.e., inactive in terms of exercise training and job, < 150 min/week moderate-intensity exercise, and no structured physical activity for 6 months prior to recruitment); no cardiopulmonary abnormalities; no injuries; the ability to pass the Exercise and Sport Science Australia (ESSA) pre-exercise screening tool and/or obtain general practitioner clearance to exercise; and the ability to ride a stationary cycle at high intensity. Exclusion criteria were known cardiovascular disease or diabetes mellitus; major or chronic illness that impairs mobility and/or eating/digestion; taking prescription medications (i.e., beta-blockers, anti-arrhythmic drugs, statins, insulin sensitizing drugs, or drugs that increase the risk of bleeding [anticoagulants, antiplatelets, novel oral anticoagulants, nonsteroidal anti-inflammatory drugs, selective norepinephrine reuptake inhibitors, or selective serotonin reuptake inhibitors]; or known bleeding disorders (i.e., hemophilia A [factor VIII deficiency], von Willebrand disease, or other rare factor deficiencies including I, II, V, VII, X, XI, XII, and XIII).

### Participant Baseline Measurements, Exercise Testing, and Familiarization

Participants arrived at the laboratory (Melbourne, Australia) following a 10–12 h overnight fast for dual-energy X-ray absorptiometry (DXA)-based body composition analysis (Lunar iDXA; GE HealthCare, Chicago, IL, USA). Upon arrival each participant’s height and body mass were recorded, bladder was voided, and any metal jewelry or clothing items containing metal were removed prior to DXA scanning. Next, resting metabolic rate (RMR) testing was performed using a calibrated TrueOne 2400 (Parvo Medics, Sandy, UT, USA) with expired air collected for a total of 25 min, including a 10 min baseline measurement and 15 min data collection. Following RMR, resting blood pressure and heart rate (HR) were recorded in a seated position.

Following baseline measurements, each participant completed an incremental fitness test to volitional fatigue on an electronically braked cycle ergometer (Lode Excalibur Sport; Lode, Groningen, the Netherlands) to determine $\dot{V}{\text{O}}_{2} {\text{peak}}$ and maximal aerobic power (MAP). During the maximal exercise capacity test, expired gas was collected every 30 s via open-circuit respirometry (TrueOne 2400; Parvo Medics) with continuous HR monitoring (Polar Heart Rate Monitor; Polar Electro, Kempele, Finland). Before each test, gas analyzers were calibrated with commercially available gases (16% O_2_, 4% CO_2_), and volume flow was calibrated using a 3 L syringe. Following a 5 min warm-up at 1 W/kg, resistance was increased by 25 W every 150 s until volitional fatigue, determined as the inability to maintain a cadence > 60 rpm. Individual ${\dot{{V}}}$O_2_peak and MAP were determined, with MAP calculated as Wfinal + (*t*/150 × 25) if the final stage was not completed, to calculate the work rate for subsequent work (67.9 ± 10.2 kJ) and duration (10 min) matched HIIT and MICT exercise trials. At least 72 h prior to the first randomized exercise trial, participants returned to the laboratory for exercise trial familiarization. Following a 5 min warm-up at 1 W/kg, participants completed two cycling-based exercise sessions consisting of a single bout of HIIT and MICT (10 min total each) to confirm their ability to successfully complete exercise trials at the prescribed intensities.

### Dietary Control and Standardized Meals

Participants recorded dietary information using the Easy Diet Diary mobile phone application [18]. Food and fluid intake for all meals and snacks was recorded over a 3-day period and analyzed by an accredited research dietician. The habitual diet record and baseline DXA/RMR data were used to prescribe a standardized meal, which was provided with cooking instructions and consumed at the participant’s home for dinner between 18:00–20:00 h before each trial day. The macronutrient composition of the standardized dinner was 50% carbohydrate, 30% fat, and 20% protein. Participants refrained from consuming any other food or fluids other than water from 20:00 h the evening prior to each trial. Participants also refrained from caffeine consumption after 12:00 h and alcohol consumption and ibuprofen 24 h prior to each trial.

### Exercise Trials and Skeletal Muscle Biopsy Collection

In a randomized crossover design, participants were assigned their first exercise trial (i.e., HIIT or MICT, with half of participants randomly assigned to perform the HIIT session first) prior to commencing trial days and did not perform any exercise in the 72 h prior to each trial day. The HIIT session consisted of 10 min total cycling with 1-min intervals at 85 ± 0.1% of individual MAP (176 ± 34 W) interspersed with 1-min active recovery intervals at 50 W. The MICT protocol was work- and duration-matched and consisted of an acute bout of continuous cycling at 55 ± 2% of individual MAP (113 ± 17 W). A schematic of the overall study design is shown in Fig. 1A. The two HIIT and MICT exercise trials were separated by ≥ 10 days, and it was not possible to blind participants nor the principal researchers to the order of these trials. All trials were completed between June 2019 and November 2019.

On trial days participants arrived at the laboratory at 07:00–08:00 h, having fasted overnight since consuming the standardized dinner the evening prior, and only consumed water during the trials. Each participant’s preferred arm was cannulated for blood collections (detailed below) and local anesthetic (1% lignocaine hydrochloride in saline; McFarlane; Surrey Hills, Victoria Australia; 11037-AS) was administered to the vastus lateralis by a highly experienced medical doctor. A percutaneous skeletal muscle biopsy was collected at rest prior to commencing exercise (0 min) using a Bergstrom needle modified with suction and immediately snap-frozen, placed in liquid nitrogen and stored at − 80 °C until analysis. Additional skeletal muscle biopsy samples were collected from each participant mid-exercise (5 min) and immediately post-exercise (10 min), with the total 10 min cycling duration consistent for each exercise intensity. All three biopsies for each exercise trial were taken from the same leg, with each subsequent biopsy collected 3–5 cm distal to the previous biopsy(ies). For the HIIT trial, the mid-exercise biopsy was collected during an active recovery interval on a bed placed directly behind the cycle ergometer (~ 30 s), and participants re-commenced cycling immediately after the biopsy was collected. For the MICT trial, participants stopped cycling (~ 30 s) for the mid-exercise biopsy collection and re-commenced cycling immediately after the biopsy was collected. HR (Polar Heart Rate Monitor; Polar Electro) and rating of perceived exhaustion (RPE; Borg RPE Scale out of 20) were recorded at 1 min (i.e., following completion of the first HIIT “on” interval), 5 min, and 10 min during each participant’s HIIT and MICT trials.

### Blood Sampling and Analyses

Upon arrival to the laboratory, a cannula (22 G; Terumo, Tokyo, Japan) was inserted into an antecubital vein of each participant. Two vacutainers of venous blood (6 mL each) were collected via cannula at the same timepoints as skeletal muscle biopsies above, including pre-exercise (0 min), mid-exercise (5 min), and immediately post-exercise (10 min). Lipid panels (Roche Diagnostics, Basel, Switzerland; 6380115190) including triglycerides, total cholesterol, high-density lipoproteins (HDL) and low-density lipoproteins (LDL) were immediately measured from an aliquot of whole blood (~ 19 μL) using the COBAS b 101 system (Roche Diagnostics). Following inversion ten times, one EDTA-coated vacutainer collected for plasma (Interpath, Somerton, Victoria, Australia; 454036) was immediately placed on ice, centrifuged at 1500*g* for 10 min at 4 °C, aliquoted, and stored at − 80 °C until analysis. Simultaneous measurement of glucose and lactate from plasma aliquots was performed in duplicate using a calibrated YSI 2900 Biochemistry Analyzer (YSI Incorporated, Yellow Springs, OH, USA).

### Immunoblotting

Snap-frozen human skeletal muscle biopsy samples were lysed in homogenization buffer containing 50 mM Tris–HCl (pH 7.5), 1 mM ethylenediaminetetraacetic acid (EDTA), 1 mM egtazic acid (EGTA), 10% glycerol, 1% Triton-X, 50 mM sodium fluoride, 5 mM sodium pyrophosphate with cOmplete Protease Inhibitor Cocktail and PhosSTOP phosphatase inhibitor (Sigma-Aldrich, St. Louis, MO, USA). Samples were centrifuged at 16,000*g* for 30 min at 4 °C and the protein content of the supernatant was determined using bicinchoninic acid (BCA) assay (Pierce, Rockford, IL, USA). Lysates (10 µg protein per well) suspended in Laemmli sample buffer were run on 4–15% pre-cast stain-free gels (Bio-Rad, Hercules, CA, USA) and transferred to polyvinylidene fluoride (PVDF) membranes (Merck Millipore, Burlington, MA, USA). Membranes were blocked with 7.5% bovine serum albumin (BSA) in Tris-buffered saline containing 0.1% Tween 20 (TBS-T) for 1 h at room temperature then incubated with primary antibodies overnight with rocking at 4 °C. After washing with TBS-T, membranes were incubated with secondary antibody for 1 h at room temperature. Antibodies against phospho-AMPK T172 (2535S), phospho-ACC S79 (11818S), and horseradish peroxidase-conjugated anti-rabbit (7074) IgG secondary antibodies were purchased from Cell Signaling Technology (Danvers, MA, USA). Proteins were detected via chemiluminescence using Bio-Rad Clarity Western ECL Substrate and imaged using the ChemiDoc Imaging System (Bio-Rad). The volume density of each target band was quantified using Bio-Rad Image Lab and normalized to total protein in each lane using stain-free imaging technology and Image Lab software (version 6.1, Bio-Rad).

### Phosphoproteomic Sample Preparation

**Fig. 2:**
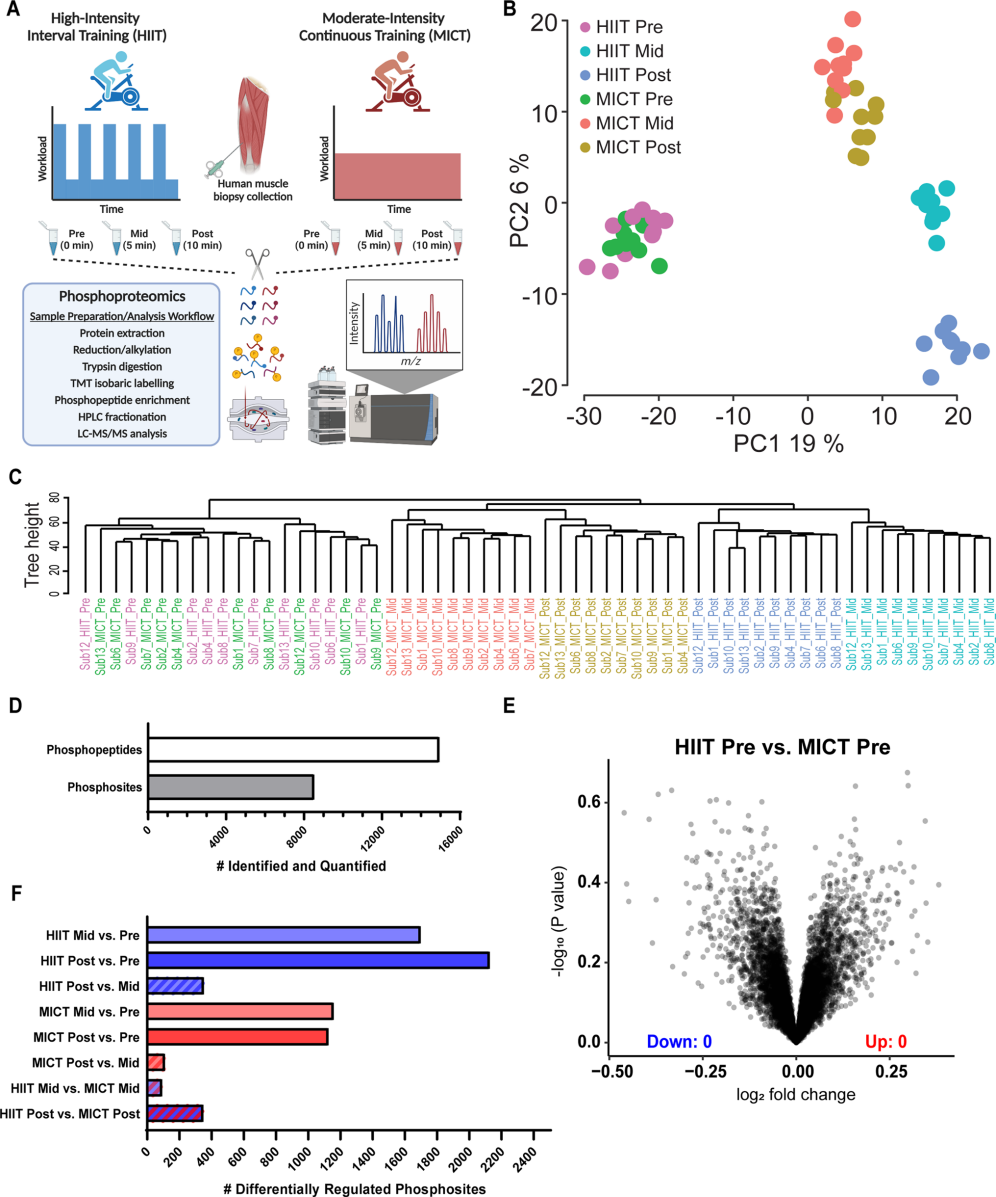
Human skeletal muscle phosphoproteomic analysis reveals effective pre-exercise standardization and distinct signaling profile clusters in response to HIIT versus MICT after 5 min and 10 min. Vastus lateralis skeletal muscle biopsies were collected pre-exercise (0 min), mid-exercise (5 min), and immediately post-exercise (10 min) from each participant during the HIIT and MICT exercise trials (**A**). The 10 min HIIT cycling session consisted of alternating 1 min intervals at 85 ± 0.1% of individual MAP (176 ± 34 W) and 1 min active recovery intervals at 50 W. The total duration- and work-matched MICT cycling session consisted of 10 min cycling at 55 ± 2% of individual MAP (113 ± 17 W). Each of the 60 total muscle biopsy samples were prepared and subjected to LC–MS/MS analysis to accurately identify and quantity skeletal muscle protein phosphorylation sites at 0 min (pre-exercise), 5 min (mid-exercise), and 10 min (post-exercise) for both the HIIT and MICT exercise trials (**A**). Principal component analysis (**B**) and hierarchical clustering (**C**) of the phosphoproteomic datasets resulting from LC–MS/MS analysis of the 60 muscle biopsy samples were performed using the PhosR phosphoproteomic data analysis package (Kim et al. 2021 *Cell Reports*). Each individual point (**B**) or line (**C**) represents a unique biological sample, and samples are color-coded by exercise intensity and timepoint. Overall, 19% of the total variance in the overall phosphoproteomic dataset was explained by principal component (PC)1, while PC2 explained 6% of the variance. The total number of phosphopeptides and phosphosites identified and quantified using MS are shown (**D**), in addition to the number of differentially regulated phosphosites (± 1.5-fold change and adjusted *P* < 0.05) from each timepoint and/or exercise intensity comparison (**F**). Volcano plot shows the median phosphopeptide log_2_ fold change (*x*-axis) plotted against the − log_10_ *P*-value (*y*-axis) for each pre-exercise condition, with no differentially regulated phosphosites at rest between crossover trials (**E**)

As depicted in Fig. 2A and detailed below, proteins from each human skeletal muscle biopsy sample were extracted, digested to peptides with trypsin, and isobarically labeled prior to phosphopeptide enrichment, fractionation, and analysis by liquid chromatography with tandem mass spectrometry (LC–MS/MS). Briefly, ~ 30 mg of each snap-frozen human skeletal muscle was lysed as previously described [17] in 6 M guanidine HCL (Sigma, St. Louis, MO, USA; G4505) and 100 mM Tris pH 8.5 containing 10 mM tris(2-carboxyethyl)phosphine (Sigma; 75259) and 40 mM 2-chloroacetamide (Sigma; 22790) using tip-probe sonication. The resulting lysate was heated at 95 °C for 5 min and centrifuged at 20,000*g* for 10 min at 4 °C, and the resulting supernatant was diluted 1:1 with water and precipitated with 5 volumes of acetone at − 20 °C overnight. Lysate was then centrifuged at 4000*g* for 5 min at 4 °C and the protein pellet was resuspended in Digestion Buffer (10% 2,2,2-trifluoroethanol [Sigma; 96924] in 100 mM HEPES pH 8.5). Protein content was determined using BCA (Thermo Fisher Scientific, Waltham, MA, USA). Four hundred μg protein (normalized to 100 μL final volume in Digestion Buffer) was digested with sequencing grade trypsin (Sigma; T6567) and LysC (Wako Chemicals, Richmond, VA, USA; 129-02541) at a 1:50 enzyme:substrate ratio at 37 °C overnight with shaking at 2000 rpm.

Digested peptides were labeled with 800 μg of 10-plex tandem mass tags (TMT) in 50% acetonitrile to a final volume of 200 μL at room temperature for 1.5 h. The TMT reaction was deacylated with 0.3% (*w*/*v*) of hydroxylamine for 10 min at room temperature and quenched to final volume of 1% trifluoroacetic acid (TFA). Each experiment consisting of seven TMT labeled peptides (ten total experiments, each including all six timepoints from a single participant’s HIIT and MICT trials and a pooled internal reference mix of peptides consisting of peptides from all ten participants) was then pooled, resulting in a final amount of 4 mg peptide per TMT 10-plex experiment. The sample identity and labeling channels have been uploaded as a table with the raw proteomic data to the ProteomeXchange Consortium via the PRIDE partner repository (see Resource Availability for login details).

In total, 20 μg of TMT-labeled peptide was removed for total proteome analysis (data not shown, as only 5–10 min of exercise does not affect total muscle protein content) and phosphopeptides were enriched from the remaining digestion of pooled peptides from each experiment using a modified version of the EasyPhos protocol [19]. Briefly, samples were diluted to a final concentration of 50% isopropanol containing 5% TFA and 0.8 mM KH_2_PO_4_. Dilutions were then incubated with 15 mg of TiO_2_ beads (GL Sciences, Tokyo, Japan; 5010–21315) for 8 min at 40 °C with shaking at 2000 rpm. Beads were washed four times with 60% isopropanol containing 5% TFA and resuspended in 60% isopropanol containing 0.1% TFA. The bead slurry was transferred to in-house packed C8 microcolumns (3 M Empore; 11913614) and phosphopeptides were eluted with 40% acetonitrile containing 5% ammonium hydroxide. The enriched phosphopeptides and 20 μg aliquot for total proteome analysis were acidified to a final concentration of 1% TFA in 90% isopropanol and purified by in-house packed SDB-RPS (Sigma; 66886-U) microcolumns. The purified peptides and phosphopeptides were resuspended in 2% acetonitrile in 0.1% TFA and stored at − 80 °C prior to offline fractionation using neutral phase C18BEH HPLC as previously described [17].

### LC–MS/MS Data Acquisition and Processing

Peptides were analyzed on a Dionex 3500 nanoHPLC, coupled to an Orbitrap Eclipse mass spectrometer (Thermo Fisher Scientific) via electrospray ionization in positive mode with 1.9 kV at 275 °C and RF set to 30%. Separation was achieved on a 50 cm × 75 μm column (PepSep, Marslev, Denmark) packed with C18-AQ (1.9 μm; Dr Maisch, Ammerbuch, Germany) over 120 min at a flow rate of 300 nL/min. The peptides were eluted over a linear gradient of 3–40% Buffer B (Buffer A: 0.1% formic acid; Buffer B: 80% acetonitrile, 0.1% v/v FA) and the column was maintained at 50 °C. The instrument was operated in data-dependent acquisition (DDA) mode with an MS1 spectrum acquired over the mass range 350–1550 *m*/*z* (120,000 resolution, 1 × 10^6^ automatic gain control [AGC] and 50 ms maximum injection time) followed by MS/MS analysis with a fixed cycle time of 3 s via HCD fragmentation mode and detection in the orbitrap (50,000 resolution, 1 × 10^5^ AGC, 150 ms maximum injection time, and 0.7 *m*/*z* isolation width). Only ions with charge state 2–7 triggered MS/MS with peptide monoisotopic precursor selection and dynamic exclusion enabled for 30 s at 10 ppm.

DDA data were searched against the UniProt human database (June 2020; UP000005640_9606 and UP000005640_9606_additional) with MaxQuant v1.6.7.0 using default parameters with peptide spectral matches, peptide, and protein false discovery rate (FDR) set to 1% [20]. All data were searched with oxidation of methionine set as a variable modification and cysteine carbamidomethylation set as a fixed modification. For analysis of phosphopeptides, phosphorylation of serine, threonine, and tyrosine was set as a variable modification, and for analysis of TMT-labeled peptides, TMT was added as a fixed modification to peptide N-termini and lysine. First search MS1 mass tolerance was set to 20 ppm followed by recalibration and main search MS1 tolerance set to 4.5 ppm, while MS/MS mass tolerance was set to 20 ppm. MaxQuant output data were initially processed with Perseus [21] to remove decoy data, potential contaminants, and proteins only identified with a single peptide containing oxidized methionine. The “expand site” function was additionally used for phosphoproteomic data to account for multi-phosphorylated peptides prior to statistical analysis.

### Bioinformatic Analysis

For analysis of human muscle phosphopeptides with TMT-based quantification, data were first log_2_ transformed and each phosphosite abundance was corrected by subtracting the abundance of the pooled sample in the same TMT batch. The phosphoproteomic data were processed using the pipeline implemented in the PhosR package [22]. Filtering was performed to retain phosphosites present in at least three participants (out of ten total participants), in at least one timepoint (out of six total timepoints). Missing values in the retained phosphosites were imputed first by a site- and sample condition-specific imputation method, where for a phosphosite that contains missing values in a condition, if more than three samples were quantified in that condition, the missing values were imputed on the basis of these quantified values for that phosphosite in that condition, and then by a random-tail imputation method [23]. The imputed data were normalized using the “combat” function in the sva package [24] for removing batch effects and then the “RUVphospho” function in PhosR for the removal of additional unwanted variation with a set of stably phosphorylated sites as negative controls [25]. The batch-corrected data were further converted to ratios relative to the pre-exercise samples (i.e., “0 min” controls). Baseline reliability and variability of phosphosite quantification between trials were assessed using the intraclass correlation coefficient (ICC) and coefficient of variation (CV). CV was calculated as SD/|mean|× 100, and ICC was computed using a two-way random effects model, measuring absolute agreement between conditions (Supplementary Table 4). Reproducibility classification of ICC values followed Cicchetti’s thresholds (i.e., excellent ≥ 0.75, good 0.60–0.74, fair 0.40–0.59, and poor < 0.40) [26].

**Fig. 3:**
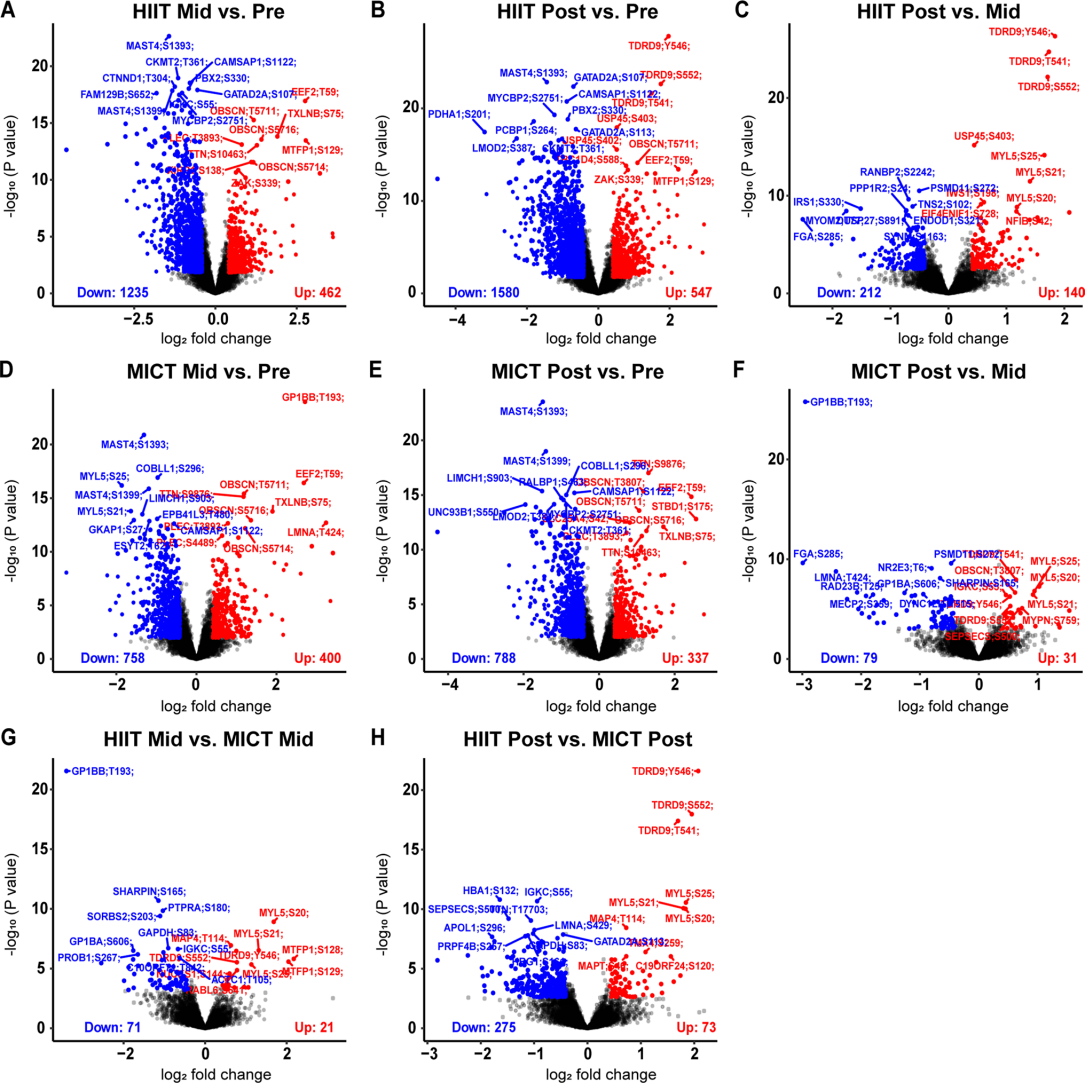
Human skeletal muscle phosphorylation sites differentially regulated by an acute bout of work- and duration-matched HIIT and/or MICT. Volcano plots showing the median phosphopeptide log_2_ fold change (*x*-axis) are plotted against the − log_10_ *P* value (*y*-axis) for each individual exercise intensity versus the respective timepoint (**A**–**F**). From the ~ 15,000 total phosphopeptides detected, significantly up-regulated (red dots) and down-regulated (blue dots) phosphosites are shown (± 1.5-fold change and adjusted *P* < 0.05), with black dots representing phosphosites that were detected but not significantly regulated by exercise. Volcano plots comparing signaling responses with each exercise intensity (i.e., HIIT versus MICT) at each timepoint are shown in **G**, **H**

**Fig. 4:**
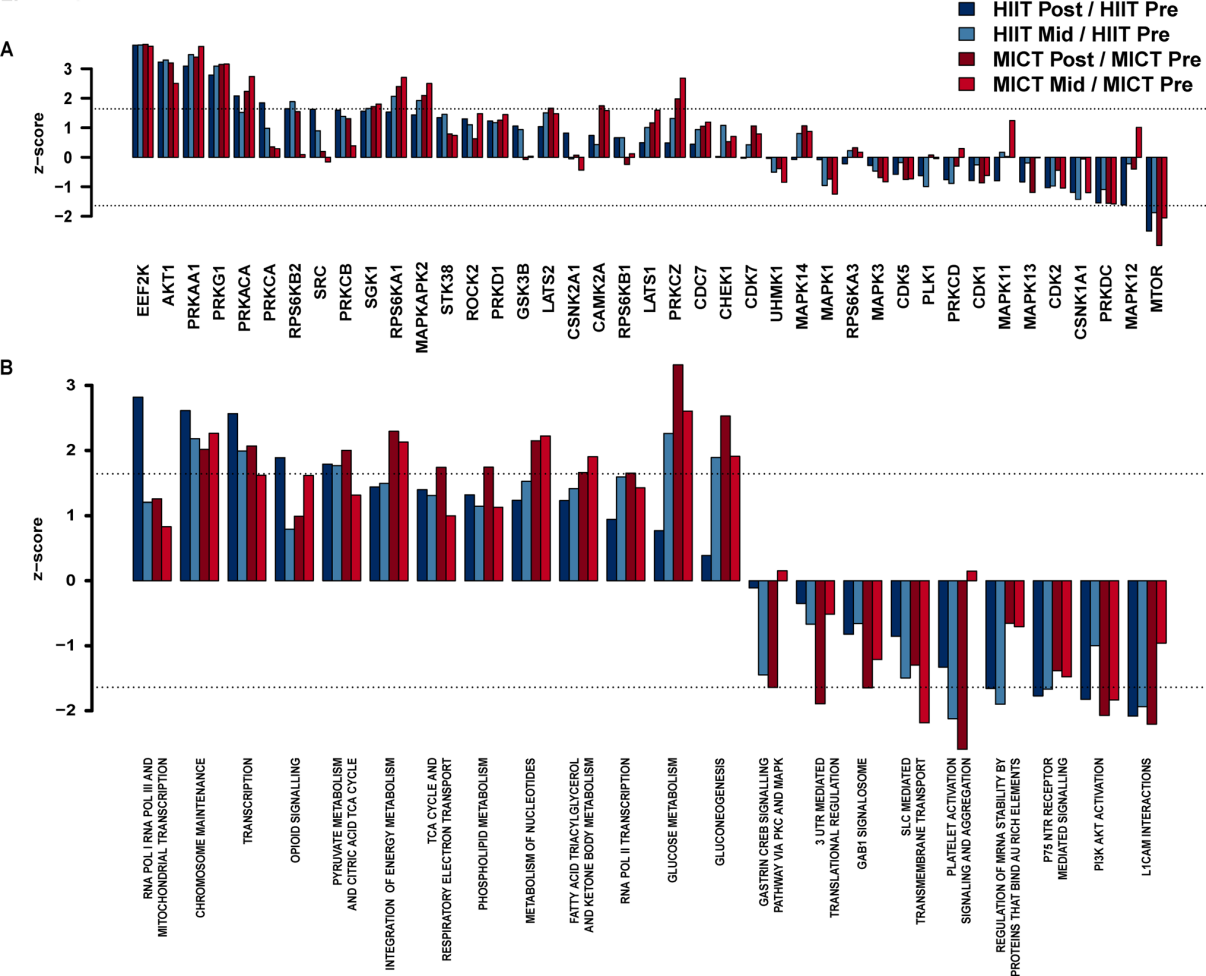
Kinase and pathway enrichment uncovers common and unique kinases and pathways regulated by HIIT and/or MICT. Kinase activity (**A**) was inferred via direction analysis using kinase perturbation analysis (KinasePA; [28]) to annotate and visualize how kinases and their known substrates are perturbed by each exercise intensity and timepoint. Pathway enrichment analysis (**B**) was performed using the Reactome database [30] to determine biological pathways that are enriched within the lists of significantly regulated genes (corresponding to their respective phosphoproteins) for each exercise intensity and timepoint relative to its respective pre-exercise control. For kinase activity inference (**A**) and pathway enrichment (**B**), *z*-scores above and below the dotted lines (corresponding to |*z*-score|> 1.64) were considered as increased or decreased by exercise, respectively, as they correspond to a one-tailed *P* value of ~ 0.05 in normally distributed data

Differentially phosphorylated sites were identified using the limma R package [27]. Phosphosites with ± 1.5-fold change and FDR-adjusted *P* value < 0.05 from each timepoint and exercise intensity comparison were considered as differentially phosphorylated (Fig. 3A–H). Kinase activities at post- or mid-exercise for both HIIT and MICT were inferred on the basis of the changes in phosphorylation (relative to the corresponding pre-exercise control samples) of their known substrates using the KinasePA package [28] and the PhosphoSitePlus annotation database [29] (Fig. 4A). Pathway enrichment analysis was then performed, whereby phosphosites were first summarized into their host protein levels by taking the maximum log_2_ fold change for each comparison between conditions, and then the pathway enrichment was performed on the basis of the inferred host protein changes using the KinasePA package and Reactome database [30] (Fig. 4B).

**Fig. 5:**
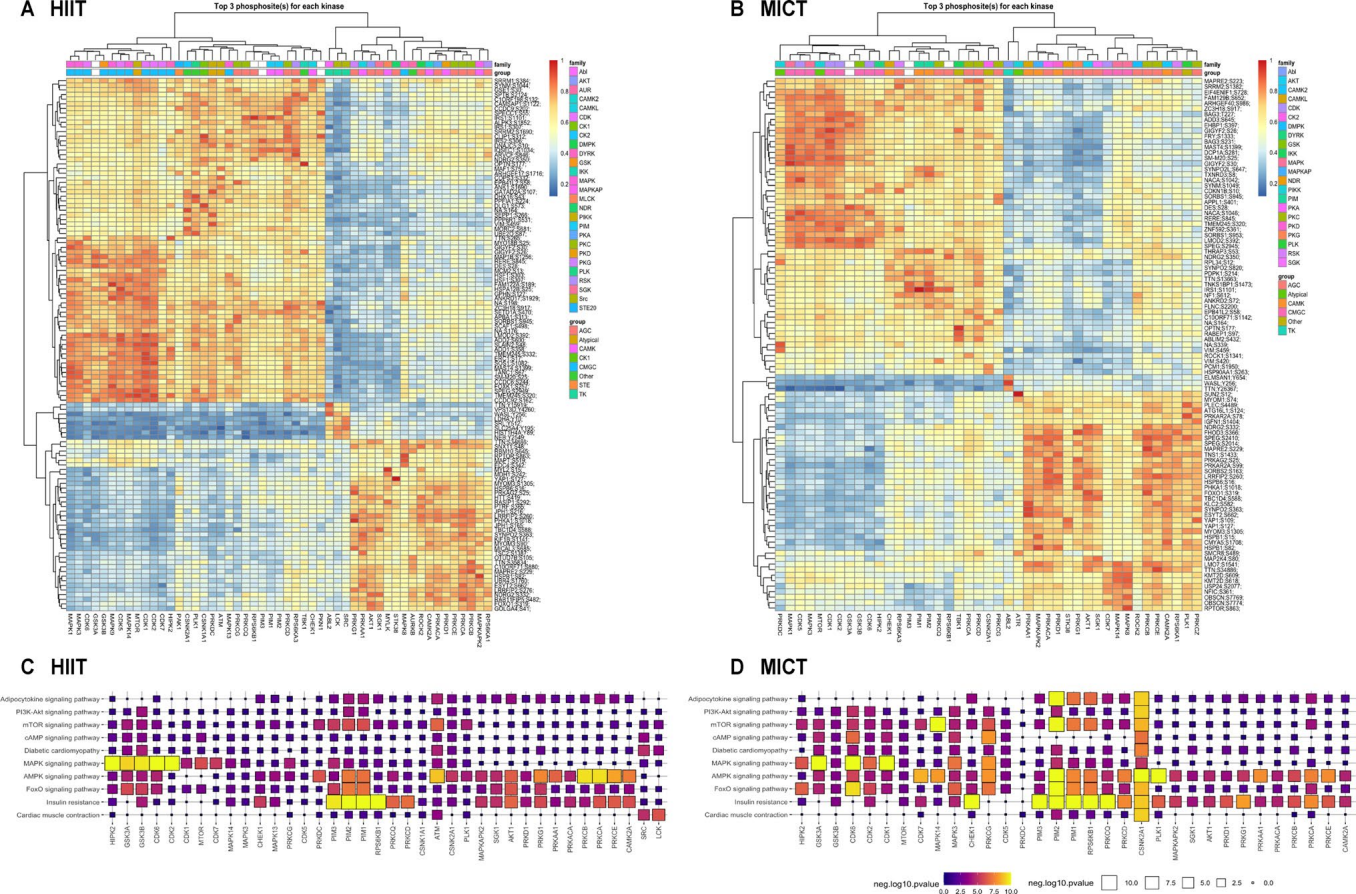
Kinase–substrate predictions and pathway enrichment analysis identify differential regulation of downstream substrates and pathways in response to HIIT versus MICT. Kinase–substrate associations were predicted in response to HIIT and MICT via the phosphoproteome signaling profiles and kinase recognition motif of known substrates using PhosR [22]. This analysis generated prediction matrices, with columns corresponding to kinases, rows corresponding to phosphosites, and values in the heatmaps denoting how likely a phosphosite is phosphorylated by a given kinase in response to HIIT (**A**) and MICT (**B**). Pathway enrichment analysis was performed using kinase–substrate predictions (i.e., phosphosites with a high prediction score for each kinase) to determine how kinases regulate common and/or distinct signaling pathways in response to HIIT (**C**) and MICT (**D**)

**Fig. 6:**
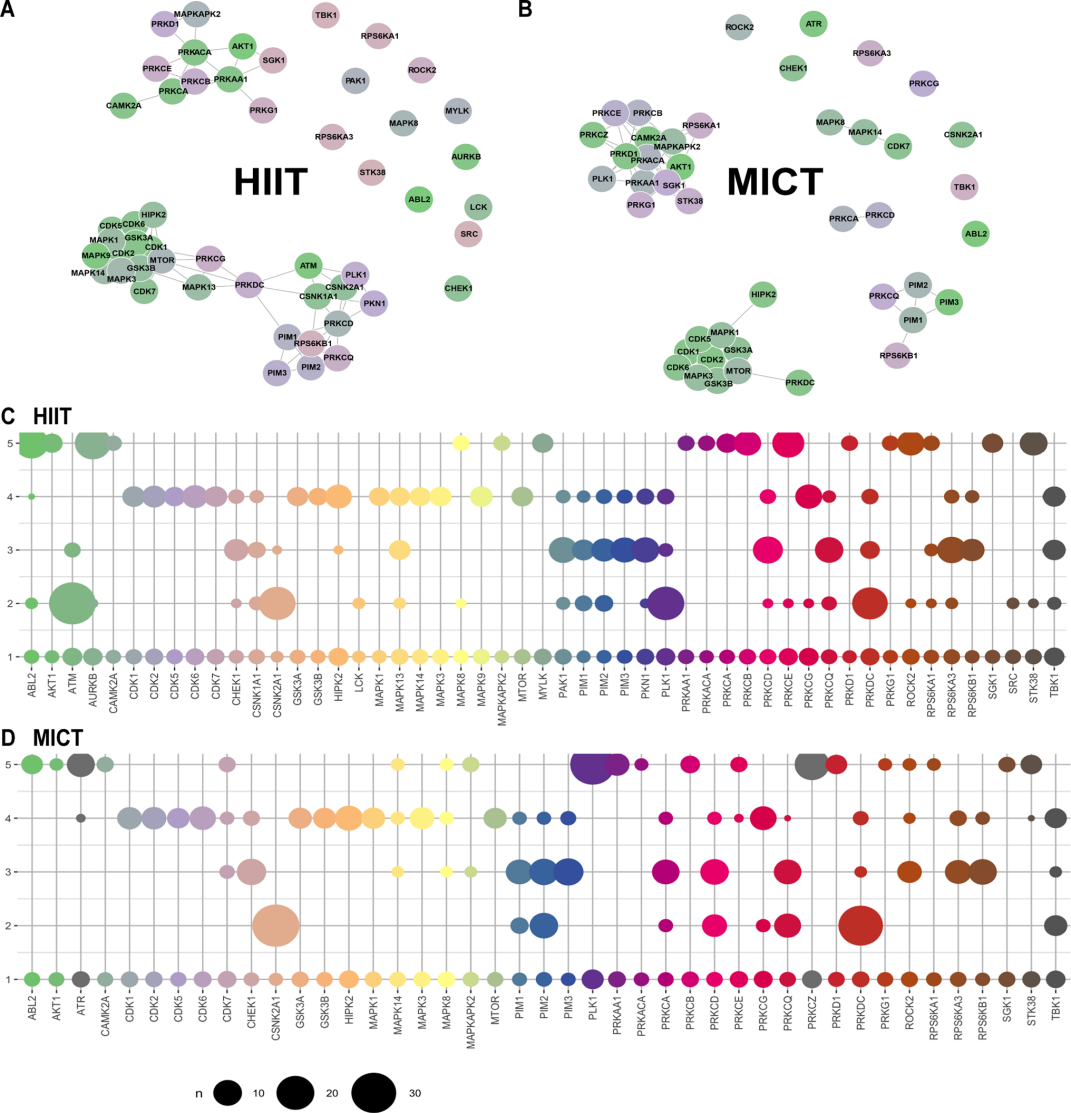
Signalome network highlights distinct HIIT and MICT kinase clusters and differential signaling trajectories in response to each exercise intensity. Signalome networks for HIIT (**A**) and MICT (**B**) exercise were constructed using the PhosR phosphoproteomic data analysis package [22]. This “signalome” construction method utilized the phosphoproteome signaling profile and kinase recognition motif of known substrates to visualize the interaction of kinases and their collective actions on signal transduction. Kinases clustered together are highly correlated in terms of kinase–substrate predictions. Visualization of five phosphoprotein clusters from the phosphoproteomic dataset highlights distinct kinase–substrate regulation within the HIIT and MICT signaling networks, with shared and unique signaling trajectories shown for a panel of kinases in response to HIIT (**C**) and MICT (**D**)

Putative substrates of kinases for HIIT and MICT were predicted using the “kinaseSubstrateScore” function in the PhosR package, and the results were represented as heatmaps (Fig. 5A, B). Pathway over-representation analysis was performed on protein sets identified from the kinase–substrate scoring analysis (i.e., kinase–substrate pairs with a score > 0.85 were selected) per kinase using the “enrichKEGG” function implemented in the clusterProfiler R package [31], and *P* values were adjusted for multiple testing using Benjamini–Hochberg FDR correction at *α* = 0.05 (Fig. 5C, D). The prediction scores were subsequently used for constructing signalome networks. Pearson’s correlation was performed on pairwise kinases, and then the correlation matrix was binarized on the basis of the correlation score threshold of 0.85. Undirected graphs were built from the binary adjacency matrix using the “graph_from_adjacency_matrix” function from the igraph package [32], and results from this analysis were presented as network diagrams (Fig. 6A, B).

Five protein modules were identified by clustering proteins with phosphosites sharing similar dynamic phosphorylation profiles and kinase regulation across both HIIT and MICT. The proportion of phosphosites that were phosphorylated by kinases for each protein module was calculated and presented as bubble plots (Fig. 6C, D). The activity of each protein module was then inferred. The regulated phosphosites were first obtained across all conditions (i.e., analysis of variance [ANOVA] test with adjusted *P* < 0.05) and the average log_2_ fold change of the regulated phosphosites for each of the five modules (relative to the corresponding pre-exercise control) were calculated (Supplementary Fig. 2A–E).

### Statistical Analysis

**Fig. 7:**
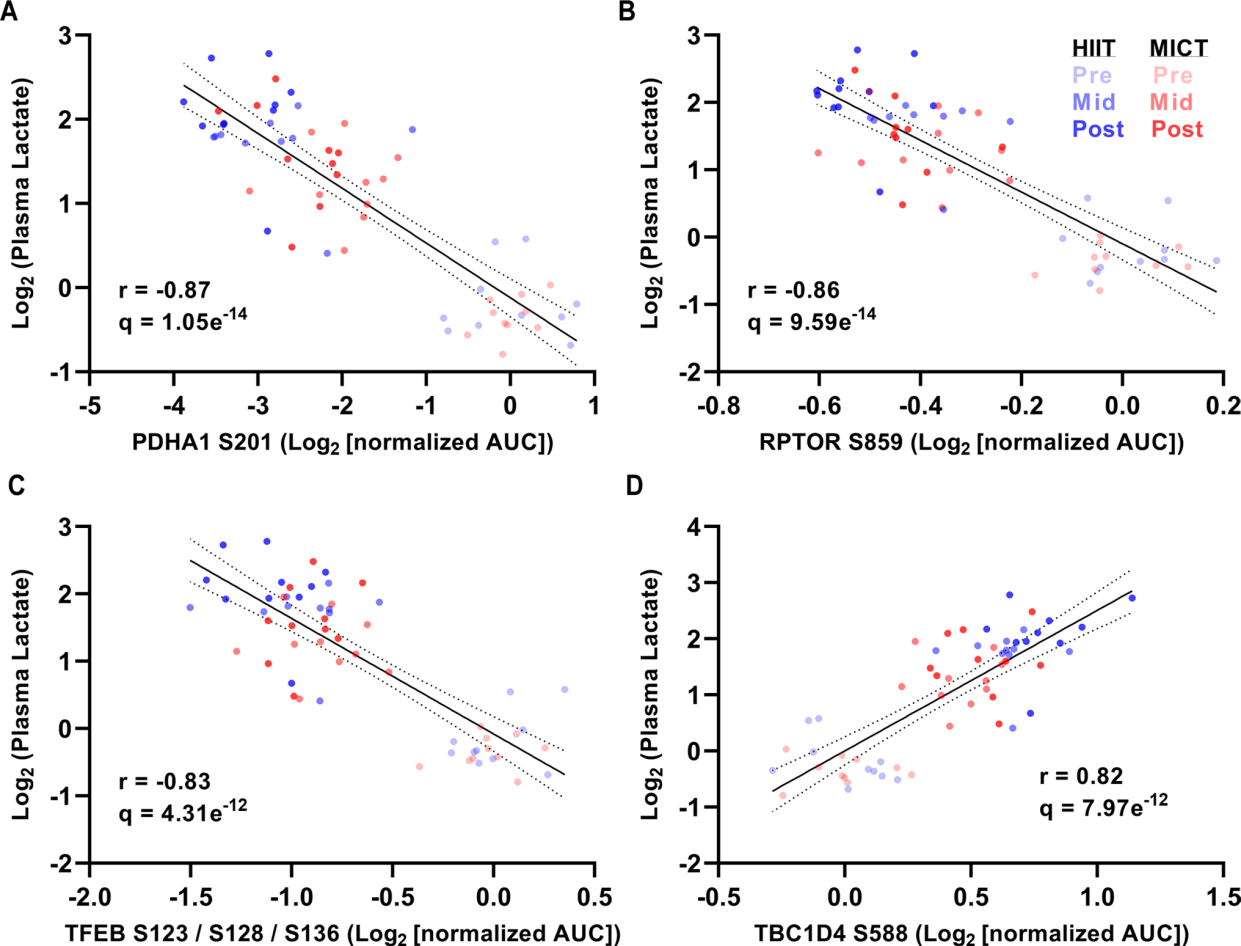
Correlation of HIIT and MICT phosphosites and plasma lactate levels identifies > 3000 lactate-correlated sites including functional phosphosites that govern protein activity and metabolic regulation. Spearman correlation of individual phosphorylation sites (*n* = 8509 total) with plasma lactate concentrations at each timepoint and exercise intensity (*n* = 60 total plasma samples analyzed) are shown for four of the most significantly correlated sites (*q* < 0.05 with Benjamini–Hochberg FDR) with annotated functional roles in governing their respective phosphoprotein’s activation state and regulating a range of key metabolic processes (e.g., glycolysis, glucose transport, and mitochondrial biogenesis) including PDHA1 S201 (**A**), RPTOR S859 (**B**), TFEB S123/S128/S136 (**C**), and TBC1D4 S588 (**D**)

Statistical analysis was performed using GraphPad Prism (version 9.4). A two-way ANOVA with repeated measurements was used to determine the effects of time and exercise intensity, with Tukey’s test applied for multiple comparisons (*P* < 0.05 considered as significant; sample size and statistical parameters are reported in the Fig. 1 legend). Spearman correlation of individual phosphorylation sites with plasma lactate concentrations at each timepoint and exercise intensity was performed to determine significantly correlated phosphosites, with Benjamini–Hochberg false discovery rate applied (*q* < 0.05 considered as significant; sample size and statistical parameters are reported in the Fig. 7 legend).

## Results

Ten healthy male participants were recruited and successfully completed all preliminary testing and the HIIT and MICT exercise bouts. The randomized crossover trial design permitted signaling responses to workload- and duration-matched HIIT and MICT exercise to be mapped and interrogated within the same participant (Fig. 1A). Baseline whole-body anthropometric measurements and blood analyses confirmed these participants (age 25.4 ± 3.2 years; BMI 23.5 ± 1.6 kg/m^2^) were metabolically healthy, and maximal exercise capacity testing confirmed they were untrained (relative peak oxygen uptake [${\dot{{V}}}$O_2_peak] 37.9 ± 5.2 mL/kg/min) in line with our recruitment strategy to maximize detection of skeletal muscle signaling responses to exercise (Fig. 1B). MAP (208 ± 40 W) achieved during the incremental fitness test to volitional fatigue was used to prescribe the relative work-matched intensities for HIIT and MICT exercise. Lean mass from the DXA scan and resting metabolic rate (Fig. 1B) were used to prescribe a standardized meal for each participant to consume prior to each exercise trial day.

Following the consumption of a standardized dinner the evening before each trial, blood and skeletal muscle biopsy samples were collected in the fasted state at baseline and at 5 min and 10 min of each respective exercise bout (Fig. 1A). Participants completing the acute bout of HIIT cycling, which consisted of 5 × 1 min work bouts at 85 ± 0.1% of individual MAP (176 ± 34 W) with 5 × 1-min active recovery intervals at 50 W, displayed increased plasma lactate concentrations at 5 and 10 min of exercise relative to pre-exercise baseline (Fig. 1C; *P* < 0.0001 and *P* < 0.001, respectively; main effect of time *P* < 0.0001) with no changes in plasma glucose levels (Fig. 1D). In response to a total work- and duration-matched acute bout of MICT (55 ± 2% individual MAP; 113 ± 17 W), participants displayed increased plasma lactate concentrations at 5 and 10 min of exercise compared with baseline (Fig. 1C; *P* < 0.001; main effect of time *P* < 0.0001) with no changes in plasma glucose concentrations (Fig. 1D). There was a main effect of higher plasma lactate levels in response to HIIT versus MICT across the exercise bout (Fig. 1C; *P* < 0.05), but no interaction effect (Fig. 1C; *P* = 0.0573). Associated increases in HR (Fig. 1E) and RPE (Fig. 1F) were observed at 5 and 10 min of HIIT and MICT relative to baseline with a main effect of time, but no HIIT versus MICT or interaction effects detected (Fig. 1E, F).

To map the signaling networks regulated by HIIT and MICT, proteins from each skeletal muscle biopsy sample were extracted, reduced/alkylated, digested to peptides, and isobarically labeled prior to phosphopeptide enrichment, fractionation, and liquid chromatography with tandem MS (LC–MS/MS) phosphoproteomic analysis (Fig. 2A). Principal component analysis (PCA; Fig. 2B) and hierarchical clustering (Fig. 2C) using the PhosR phosphoproteomic data analysis pipeline [22] revealed a high level of consistency in the overall baseline signaling signature at rest (0 min) prior to the HIIT and MICT exercise trials, confirming our pre-exercise standardization strategies were effective. Distinct clusters were observed in response to the two divergent exercise challenges, with the 5 and 10 min HIIT signaling profiles clustering farthest away from the pre-exercise control profile (Fig. 2B, C). Overall, 19% of the total variance in the phosphoproteomic dataset was explained by principal component (PC)1, while PC2 explained 6% of the variance (Fig. 2B). Global phosphoproteomics identified and quantified a total of 14,931 phosphopeptides (Fig. 2D; Supplementary Table 1), corresponding to 8509 phosphorylation sites (Fig. 2D; Supplementary Table 2). The distributions of phosphosite quantifications are shown in Supplementary Fig. 1A and detailed in Supplementary Table 2 for each of these 8509 phosphosites, with the largest proportion of sites quantified in all ten participants, and a median of 70% of samples in which sites were quantified (i.e., seven out of ten total participants).

Bioinformatic analyses of these phosphoproteomic data using PhosR [22] identified > 1000 phosphosites significantly regulated (± 1.5-fold change; adjusted *P* < 0.05; identified and quantified in ≥ 3 participants and ≥ 1 timepoint) by HIIT and/or MICT after 5 and 10 min, with < 400 of these sites differentially regulated post- versus mid-exercise (Fig. 2F; Supplementary Table 3). Analysis of the baseline signaling signatures between the two crossover trials revealed no significantly regulated phosphosites (Fig. 2E), highlighting the high level of control and reproducibility in the resting phosphoproteome between trials. Reproducibility classification of ICC values for the 8509 total quantified phosphosites showed 50% of phosphosites (4256) had excellent reproducibility at baseline between trials. The remaining 4253 phosphosites had either good (1603), fair (1281), or poor (1369) baseline reproducibility classification (Supplementary Table 4).

Significantly regulated phosphosites observed in response to HIIT and/or MICT included kinases and substrates shown to be regulated by an acute bout of continuous high-intensity cycling, such as those within the AMPK pathway ([16]; e.g., ACACB S222 [HIIT and MICT], AKAP1 S107 [MICT only], RPTOR S722 [HIIT and MICT], STBD1 S175 [HIIT and MICT]; [33]), TBC1D1 S237 [HIIT and MICT], and TBC1D4 S704 [5 min HIIT only]). Multiple substrates of protein kinase A (PKA; PHKA1 S1018, and HSBP6 S16) were increased by both HIIT and MICT, while the PKA substrate CDK16 S110 was only increased after 10 min of HIIT. In addition, the known Akt substrate IRS1 S629 was only regulated after 10 min of both HIIT and MICT [16]. Immunoblot analysis of the canonical exercise-regulated AMPK signaling pathway confirmed exercise-induced increases in phosphorylation across the HIIT and MICT exercise bouts in AMPK T172 (Supplementary Fig. 1B; main effect of time *P* < 0.01; no HIIT versus MICT or interaction effects) and downstream ACC (ACACA) S80 (Supplementary Fig. 1C; main effect of time *P* < 0.0001; main effect of HIIT versus MICT *P* < 0.01; interaction effect *P* = 0.001, with higher levels of phosphorylation in response to HIIT).

In response to HIIT, a total of 1697 phosphosites (Fig. 3A; including 1235 and 462 down- and up-regulated, respectively) and 2127 phosphosites (Fig. 3B; including 1580 and 547 down- and up-regulated, respectively) were significantly regulated after 5 and 10 min of exercise, respectively (Fig. 2F; Supplementary Table 3). The top ten significantly regulated sites displaying the most robust fold change increases in phosphorylation in response to HIIT at 5 min versus rest included MYLPF T10, ZAC S454, FGA S285, MTFP1 S129, EEF2 T59, MAPRE2 S229, LMNA T424, HRC T207, ADSSL1 S7, and MTFP1 S128. The ten phosphosites’ fold changes decreasing the most in response to HIIT at 5 min compared with rest included RTN4 S738, PDHA1 S262, DCLK1 S358, PDHA1 S201 and S269, SYNM T1109, MAP1B S1793, NGFR S217, SLC4A1 S349, and TRIP10 S354 (Fig. 3A; Supplementary Table 3). After 10 min, top phosphosite fold changes most robustly increased by HIIT versus rest included CBX1 S89, MAPRE2 S229, MTFP1 S129, HRC T207, EEF2 T59, STBD1 S175, EEF2 T57, HRC S299, ZAK S454, and CIC S1371. Sites displaying the most decreased fold changes in phosphorylation by HIIT at 10 min included RTN4 S738, PDHA1 S201, SLC4A1 S349, KRI1 S142, NGFR S217, DCLK1 S358, CMYA5 S2825, TXNIP T294, CLASP2 S326, and SRRMS S2398 (Fig. 3B; Supplementary Table 3). A total of 352 sites were differentially regulated after 10 min versus 5 min HIIT (Fig. 2F), including 212 down- and 140 up-regulated phosphosites (Fig. 3C; Supplementary Table 3).

MICT induced changes in a lower number of significantly regulated phosphosites including a total of 1158 phosphosites after 5 min (Figs. 2F and 3D; 758 and 400 down- and up-regulated, respectively) and 1125 phosphosites after 10 min (Figs. 2F and 3E; 788 and 337 down- and up-regulated, respectively). A total of 110 sites were differentially regulated after 10 min versus 5 min MICT, including 79 down- and 31 up-regulated phosphosites (Figs. 2F and 3F; Supplementary Table 3).

To identify unique sites regulated by a single bout of HIIT relative to MICT, the signaling responses to each exercise intensity were next compared. After 5 min of HIIT, 92 total phosphosites were differentially regulated, including 71 sites down-regulated and 21 sites up-regulated, relative to MICT (Figs. 2F and 3G; Supplementary Table 3). These exercise intensity-specific signaling responses to HIIT were observed to be more robust at 10 min, with 348 total phosphosites differentially regulated compared with MICT (Figs. 2F and 3H; Supplementary Table 3; 275 and 73 down- and up-regulated, respectively). Top exercise intensity-specific phosphosites most robustly up-regulated by HIIT relative to MICT in terms of fold change included MTFP1 S128/S129 (increased by 5 and 10 min HIIT; S129 also increased by 5 and 10 min MICT), MYL5 S20/S21/S25 (increased by 10 min HIIT; decreased by 5 min MICT), and TDRD9 Y546/S552 (increased by 10 min HIIT; decreased by 5 min MICT). Exercise intensity-specific phosphosites down-regulated the most by HIIT versus MICT in terms of fold change included GP1BB T193 (decreased by 5 and 10 min HIIT; increased by 5 min MICT), ATAD2B S374 (decreased by 5 and 10 min HIIT; decreased by 10 min MICT), MAPK1 T181 (decreased by 5 min HIIT; not regulated by MICT), ADD2 S532 and GP1BA S606 (decreased by 5 and 10 min HIIT; increased by 5 min MICT), and RYR1 T1399 (decreased by 5 and 10 min HIIT; decreased by 10 min MICT) (Fig. 3G, H; Supplementary Table 3).

Kinase enrichment analyses were next performed to identify common and divergent activation/deactivation of kinases in response to the two exercise bouts (Fig. 4A). Kinase activities were inferred via direction analysis using kinase perturbation analysis (kinasePA; [28]), to annotate and visualize how kinases with at least five quantified known substrates were perturbed by each exercise intensity and timepoint. These analyses confirmed activation/deactivation of known exercise-regulated kinases in response to both HIIT and MICT relative to pre-exercise, with significant kinase activity enrichments considered as kinases displaying a *|z*-score|> 1.64 (corresponding to a one-tailed *P* value of 0.05 in normally distributed data). Kinases displaying similar increases in the levels of inferred kinase activity in response to both HIIT and MICT after 5 and/or 10 min included eukaryotic elongation factor 2 kinase (EEF2K), AKT1, AMPK alpha 1 catalytic subunit (PRKAA1), protein kinase cGMP-dependent 1 (PRKG1), PKA subunit alpha (PRKACA), serum/glucocorticoid regulated kinase 1 (SGK1), ribosomal protein S6 kinase alpha-1 subunit (RPS6KA1), and MAP kinase-activated protein kinase 2 (MAPKAPK2). Moreover, exercise-induced decreases in the kinase activity of mammalian target of rapamycin (MTOR) were observed in response to both HIIT and MICT after 5 and 10 min.

Kinase enrichment analyses also revealed unique kinases that were differentially activated/deactivated (i.e., |*z*-score|> 1.64) in response to HIIT versus MICT after 5 and/or 10 min (Fig. 4A). For example, activation of unique protein kinase C (PKC) conventional/atypical isoforms were observed to be differentially regulated by HIIT versus MICT, with increased activity of the conventional PKC alpha isoform (PRKCA; *z*-score > 1.64) in response to 10 min HIIT only. In contrast, activity of the atypical PKC zeta isoform (PRKCZ) was increased only by 5 and 10 min MICT (*z*-score > 1.64). Other kinases shown to be uniquely activated by HIIT or MICT included S6 kinase beta-2 subunit (RPS6KB2; increased only by 5 and 10 min HIIT; *z*-score ≥ 1.64) and CAMK2A (increased only by 5 and 10 min MICT; *z*-score ≥ 1.64). Pathway enrichment analyses were next performed using the Reactome database to determine biological pathways that were enriched within the lists of significantly regulated genes (corresponding to their respective phosphoproteins) for each exercise intensity and timepoint relative to pre-exercise, with significant enrichment considered as pathways displaying |*z*-score |> 1.64 (Fig. 4B). Up-regulated reactome pathways (i.e., *z*-scores > 1.64) enriched in response to both HIIT and MICT after 5 and/or 10 min included expected exercise-regulated biological pathways such as “chromosome maintenance,” “transcription,” “opioid signaling,” “pyruvate metabolism and citric acid TCA cycle,” “glucose metabolism,” and “gluconeogenesis.” Down-regulated reactome pathways (i.e., *z*-scores <  − 1.64) enriched in response to both HIIT and MICT after 5 and/or 10 min included “L1CAM interactions,” “PI3K Akt activation,” and “platelet activation signaling and aggregation.”

The only reactome pathway observed to be positively enriched in response to HIIT after 5 and/or 10 min but not enriched by MICT was “RNA polymerase I and III and mitochondrial transcription” (Fig. 4B). The only two pathways observed to be negatively enriched in response to HIIT after 5 and/or 10 min but not MICT included “P75 NTR receptor mediated signaling” and “regulation of mRNA stability by proteins that bind AU rich elements.” In contrast, reactome pathways positively enriched in response to MICT after 5 and/or 10 min but not enriched by HIIT included “integration of energy metabolism,” “TCA cycle and respiratory electron transport,” “phospholipid metabolism,” “metabolism of nucleotides,” and “fatty acid triacylglycerol and ketone body metabolism.” Pathways negatively enriched in response to MICT after 5 and/or 10 min that were not enriched by HIIT included “SLC mediated transmembrane transport,” “GAB1 signalosome,” “3′ UTR mediated translational regulation,” and “gastrin CREB signaling pathway via PKC and MAPK.”

Kinase–substrate associations were next predicted in response to HIIT and MICT via the phosphoproteomic signaling profiles and kinase recognition motifs of known substrates using kinasePA (Fig. 5; [28]). This analysis generated prediction matrices, with columns corresponding to kinases, rows corresponding to phosphosites, and values denoting how likely a phosphosite is phosphorylated by a given kinase in response to HIIT (Fig. 5A) and MICT (Fig. 5B). Pathway enrichment analyses were then performed using phosphosites with a high prediction score for each kinase to observe how biological pathways containing substrate phosphosites are commonly and/or uniquely regulated by HIIT (Fig. 5C) versus MICT (Fig. 5D). Collectively, these analyses revealed that a range of kinases displayed unique patterns and/or significance levels of signaling pathway regulation in response to each exercise intensity, including HIPK2, GSK3B, CDK6, CDK2, CDK1, CDK7, MAPK14, MAPK3, CHEK1, PRKCG, CDK5, PRKDC, PIM2, PRKCQ, PRKCD, CSNK2A1, PLK1, AKT1, PRKCB, and PRKCA (Fig. 5C, D).

Using these matrices, signalome networks were then reconstructed on the basis of the correlation between kinases to visualize how kinases and substrates are regulated in response to HIIT (Fig. 6A) versus MICT (Fig. 6B). This revealed distinct signalome profiles between HIIT and MICT, with kinases clustered closer together more highly correlated in terms of their kinase–substrate predictions. To determine the proportion of common and unique substrates targeted by specific kinases in response to HIIT versus MICT, phosphoproteins were clustered into protein modules according to their prediction matrices, shown in Fig. 5A, B. This clustering revealed five unique clusters of HIIT and MICT kinase–substrate signaling profiles (Supplementary Fig. 2A–E; modules 1–5). Visualization of these five clusters for individual kinases revealed shared or unique substrate signaling trajectories in response to HIIT (Fig. 6C) versus MICT (Fig. 6D) within their unique signalome networks. For example, widely studied exercise-regulated kinases, including AKT1, CAMK2A, MAPK1, MAPK3, MTOR, and PRKAA1, displayed similar substrate clusters between exercise intensities, while other kinases, such as ABL2, MAPK14, PLK1, PRKCA, PRKCE, RPS6KA1, and STK38, regulated unique substrate clusters in response to HIIT versus MICT (Fig. 6C, D).

Finally, to leverage the global phosphoproteomic dataset and test the hypothesis that higher plasma lactate concentrations across the HIIT exercise bout associate with the skeletal muscle exercise-regulated signaling proteins that become engaged, correlation analyses were performed to interrogate individual phosphorylation site responses within the dataset relative to the plasma lactate responses to HIIT and MICT exercise. A total of 3084 phosphosites were significantly correlated with plasma lactate concentrations from each timepoint and exercise intensity (Supplementary Table 5; *q* < 0.05 with Benjamini–Hochberg false discovery rate [FDR]). Among these phosphosites, 9 of the top 50 most significantly correlated phosphosites have annotated functional roles in regulating their respective protein’s activation state in the PhosphoSitePlus annotation database (Fig. 7A–D; [29]). For example, four of these top nine phosphosites that were significantly correlated with plasma lactate have experimentally validated roles in governing their respective protein’s activity and are involved in a range of key acute and chronic exercise-regulated metabolic processes (e.g., glycolysis, glucose transport, and mitochondrial biogenesis) including PDHA1 S201 (Fig. 7A), RPTOR S859 (Fig. 7B), TFEB S123/S128/S136 (Fig. 7C), and TBC1D4 S588 (Fig. 7D). Other phosphosites among the top 10% most significantly correlated sites with plasma lactate concentrations included known AMPK-regulated sites (e.g., ACACB S222 and mitochondrial fission regulator-1 like protein [MTFR1L] S141 [34]), as well as novel phosphosites with no known functional role (e.g., DENND4C S989, a phosphoprotein present in glucose transporter GLUT4 vesicles, and MTFP1 S128/S129, a phosphoprotein involved in inner mitochondrial membrane fission/fusion) that may respectively be involved in regulating skeletal muscle glucose transport and mitochondrial network dynamics in response to an acute bout of exercise and/or exercise training.

## Discussion

For the first time, this analysis of the HIIT signaling network in human skeletal muscle has revealed the early time course of acute signaling events underlying muscle adaptive responses to work-matched HIIT versus MICT. Our global phosphoproteomic approach has revealed the rapid activation/deactivation of a complex network of common and exercise intensity-specific kinases, substrates, and pathways regulated by a single bout of HIIT versus work-matched MICT after just 5 min of exercise, which were highly correlated with the prevailing plasma lactate responses across each exercise bout. To the best of our knowledge, this is the first phosphoproteomic study of exercise signaling that has matched for workload and duration to assess divergent signaling responses between exercise intensities.

The matching of total workload and duration between exercise bouts is important and contrasts previous phosphoproteomic studies that have compared exercise signaling responses with divergent workloads of differing durations and modalities [17]. Such an approach permitted resolution of time-sensitive profiles of distinct signaling responses comparing the effects of a moderate-intensity continuous exercise challenge to a high-intensity interval-based exercise protocol within the same participants in a crossover study design, allowing sufficient recovery (i.e., > 10 days) to ensure no residual effects of the previous exercise bout. Standardized meal provision before each trial prescribed in accordance with each participant’s body composition, resting metabolic rate, and food diary records led to a highly reproducible resting “baseline” signaling signature for each participant (Fig. 2B, E), confirming a high level of control and reinforcing the importance of utilizing dietary standardization in addition to just overnight fasting to help control for potential differences in nutrient/energy availability between trials.

This study utilized a metabolically healthy, but untrained participant cohort to induce robust signaling responses, as available evidence indicates exercise-induced signaling responses in skeletal muscle, such as AMPK activation, are more pronounced in untrained relative to trained human participants [35, 36]. All ten participants were able to successfully complete both work-matched exercise trials, with the HIIT protocol eliciting higher plasma lactate concentrations compared with MICT. The two exercise intensities both provided a robust contractile stimulus that engaged a range of known exercise-regulated kinases, including activation of AMPK (regulated by AMP/ATP and ADP/ATP ratios), EEF2K (regulated by Ca^2+^/calmodulin), PKA (regulated by cAMP), and PRKG1 (regulated by NO/cGMP). Activation of AKT1 increased after 10 min (e.g., inferred via known substrate IRS1 S629), while surrogate markers of MTOR activation decreased (e.g., NDRG2 T248 decreased only after 10 min HIIT; NDRG2 S344 increased only after 5 and 10 min MICT), in response to both exercise intensities. Overall, a higher number of phosphosites were down-regulated versus up-regulated at each timepoint and exercise intensity, suggestive of acute exercise-regulated kinases becoming inhibited and/or phosphatases becoming activated early after commencing exercise.

Collectively, our phosphoproteomic datasets challenge the existing exercise physiology literature showing a lack of signaling differences in key energy stress pathways such as AMPK [12], p38MAPK, and p53 [13] in response to acute bouts of work-matched HIIT and MICT, which to date has primarily involved only targeted approaches such as immunoblotting. Specifically, phosphoproteomics has identified a wider breadth and complexity of kinase and substrate regulation, including a subset of unique HIIT- versus MICT-regulated kinases, substrates, and pathways that may have exercise intensity-specific functional significance. The unique signaling regulation by acute work-matched HIIT and MICT observed in this study supports previous theories [5, 8, 37] and targeted findings in human skeletal muscle [14] from the exercise physiology field that have suggested distinct molecular metabolic responses to interval-based exercise translate to HIIT-specific kinase and substrate signaling signatures.

For example, in this study, mitochondrial fission process 1 (MTFP1; also known as MTP18) S128/S129 were identified among the top HIIT-regulated sites, with S128 only regulated by HIIT and more robust phosphorylation of S129 observed following HIIT versus MICT. The inner mitochondrial membrane protein MTFP1 has known roles in mitochondrial fission/fusion and was previously identified as having increased abundance in rat diaphragm following endurance exercise training [38], but has not been implicated in acute exercise signaling to date. MTFP1 has recently emerged as a key regulator of liver mitochondrial and metabolic activity, with liver-specific deletion of MTFP1 in mice conferring protection against high fat diet-induced metabolic dysfunction and hepatosteatosis via upregulating oxidative phosphorylation activity and mitochondrial respiration [39]. MTFP1 has been identified as a gene target of peroxisome proliferator-activated receptor gamma coactivator 1-alpha (PGC-1α) in C2C12 myotubes [40], with its translation regulated by mTORC1 activity [41]. Human skeletal muscle MTFP1 gene expression, concomitant with PGC-1α expression, was recently shown to be reduced by leg immobilization and increased upon resumption of physical activity and resistance exercise training [42]. Collectively, this growing body of evidence suggests that the unique phosphorylation of MTFP1 in response to HIIT may represent a novel mechanism underlying exercise-regulated mitochondrial inner membrane fission and/or fusion. Further investigation of MTFP1’s potential functional roles in maintaining skeletal muscle mitochondrial networks and metabolic homeostasis is warranted.

The observed differences in work-matched HIIT versus MICT signaling responses may be due to stochastic changes or accumulation of intracellular calcium and/or other metabolites in response to fluctuating energy demands during HIIT’s repeated work-rest cycles [43], and is consistent with other studies investigating effects of acute work-matched bouts of HIIT versus MICT [13, 44]. Indeed, investigations that have determined metabolic fluctuations occurring in work-matched interval versus continuous exercise have observed a greater level of activation of kinases such as AMPK, CaMKII, and p38 MAPK, suggesting that buildup of upstream stimuli for these kinases (e.g., increased AMP/ATP and/or ADP/ATP ratios, and intracellular calcium) may trigger greater activation in response to interval-based exercise [14]. While levels of AMPK activation were similar between HIIT and MICT in this study, differential activation of PKC conventional/atypical isoforms (i.e., calcium-responsive conventional PRKCA activity only increased by HIIT, while atypical PRKCZ activity only increased by MICT) suggests intracellular calcium spikes during HIIT intervals may contribute to its unique activation of PKC isoforms versus MICT. Furthermore, activation of the calcium-responsive CAMK2A, which is rapidly activated in human muscle in response to continuous exercise [45, 46], was differentially regulated by HIIT versus MICT (i.e., CAMK2A activity only increased by MICT). This suggests differential signaling mechanisms underlying calcium homeostasis may potentially contribute to acute signaling programming of chronic skeletal muscle adaptations to high-intensity exercise such as stimulation of mitochondrial biogenesis [47].

Rates of carbohydrate oxidation are increased, while rates of fat oxidation are reduced, in response to a single bout of HIIT compared with MICT [44]. Acute HIIT increases levels of skeletal muscle glucose transport, glycogenolysis, and glycolysis, leading to accumulation of glycolytic products such as muscle lactate that can act as signaling molecules and influence exercise-regulated signaling networks within skeletal muscle, as well as be released into the bloodstream to facilitate inter-organ communication with other tissues such as the heart, liver, and brain [4, 48]. Plasma lactate responses to acute HIIT in the present study were increased relative to MICT, with > 3000 phosphosites identified as being significantly correlated with the prevailing plasma lactate concentrations. Among the most highly correlated phosphosites with plasma lactate were PDHA1 S201 (Fig. 7A) and TBC1D4 S588 (Fig. 7D), with annotated functional roles in the regulation of glycolysis and glucose transport, respectively. PDHA1 is a subunit of the PDH enzyme complex, which is regulated by pyruvate and ADP and becomes activated by calcium during exercise, serving as a key link between glycolysis and the tricarboxylic acid (TCA) cycle by converting pyruvate to acetyl-CoA [4]. TBC1D4 is a key nexus in skeletal muscle glucose transport regulation via control of glucose transporter (GLUT4) translocation in response to insulin and muscle contraction during exercise [49]. Given the strong associations (i.e., |*r*|> 0.80) observed between plasma lactate concentrations and these key regulatory phosphosites in the present study, and in light of other studies investigating signaling-metabolite correlations in response to distinct exercise modalities and workloads [17], these correlation data support the hypothesis that plasma lactate accumulation across an exercise bout associates with muscle signaling responses [8]. Collectively, differences in muscle and circulating metabolites and counterregulatory hormones in response to HIIT versus MICT may therefore influence metabolite–protein interactions during/after exercise, potentially leading to beneficial physiological adaptations. In addition to increasing plasma lactate concentrations, the higher exercise intensity of HIIT versus MICT may also lead to differential muscle fiber type recruitment that in turn influences fiber type- and/or isoform expression-specific signaling pathway responses, such as AMPK [50, 51], which were undetectable given the present study’s focus on analyzing whole muscle biopsy samples.

There are several limitations of this study that warrant further investigation in future work examining human skeletal muscle exercise signaling. First, we studied only male participants to allow us to benchmark observed signaling responses to previous studies investigating continuous exercise protocols in males. The acute responses of skeletal muscle to HIIT in female human participants remain uncharacterized and are being actively investigated in our ongoing research. While each participant in the present study underwent full exercise protocol familiarization sessions prior to their HIIT and MICT trials to help minimize potential stress responses to unfamiliar exercise (i.e., “first bout effect”), it is not clear how much of the observed signaling network response is specifically due to the response to the muscle biopsy procedure and/or stress response to the exercise exposure versus mechanisms underlying the actual adaptation to exercise [52]. The HIIT and MICT protocols utilized were selected on the basis of the feasibility of untrained participants to complete each exercise bout. As a result, 5–10 min of HIIT or MICT at the prescribed intensities may not have engaged the full repertoire of shared and exercise intensity-specific signaling pathways in muscle and may require longer exercise durations and/or greater differences in exercise intensities in future studies. For example, patterns of substrate oxidation change during more prolonged exercise and differ between sexes, which may affect signaling responses more robustly between exercise intensities and/or sexes. Additional metabolic readouts, such as muscle lactate and phosphocreatine, were not feasible in this study owing to limited remaining muscle biopsy sample following phosphoproteomic analysis, but would improve understanding of the potential differences in metabolic perturbations and associated signaling responses to acute HIIT versus MICT in future studies.

While the focus of this study was on the early phosphorylation dynamics in response to each acute exercise bout, analysis of additional recovery biopsies in the hours and days following exercise in future studies may reveal longer term adaptations in the phosphoproteome and how they translate into more chronic physiological muscle adaptations. However, the signaling responses and potential programmed muscle adaptations to an acute bout of HIIT or MICT may not be able to predict chronic training adaptations [53]. For example, recent transcriptomic and proteomic analyses of mammalian skeletal muscle have highlighted that acute changes may not be predictive of chronic exercise training-induced changes [54], such as HIIT-induced remodeling of the skeletal muscle proteome after repeated training [55, 56]. Future follow-up longitudinal studies with clearly defined clinical outcomes are warranted to determine which signaling events within these complex networks are associated with specific longer-term adaptations and health benefits in response to HIIT and/or MICT. Extensive total proteome analysis was not performed in this study, as only 5–10 min of exercise has been previously observed to not affect total muscle protein content [16]. While not analyzed, given the acute nature of the exercise bouts in this study, repeated HIIT training (i.e., six sessions per week for 2 weeks) can stimulate more robust mitochondrial adaptations (i.e., greater exercise-induced increases in citrate synthase maximal activity and mitochondrial respiration) compared with total work- and duration-matched MICT in human skeletal muscle, suggesting that exercise intensity and/or the pattern of muscle contraction may drive peripheral adaptations to exercise [57]. The amplification of human skeletal muscle signaling and mRNA responses underlying mitochondrial biogenesis in response to acute low-volume, high-intensity exercise may be due to more robust induction of metabolic stress relative to more prolonged, moderate-intensity exercise [8, 58]. In this regard, it has been speculated that accumulation of skeletal muscle intracellular AMP, ADP, calcium, and/or other metabolites such as lactate during HIIT intervals may differentially affect skeletal muscle signaling responses and adaptations to exercise, such as mitochondrial biogenesis, relative to continuous bouts of exercise [37]. However, with repeated chronic HIIT versus MICT training, exercise training volume may become more important than intensity in increasing skeletal muscle mitochondrial content over time [59].

Finally, the functional relevance of HIIT and/or MICT regulation of unique kinase activation patterns including isoform-specific PKC activation and novel HIIT-regulated phosphosites, such as MTFP1 S128/S129, remains unknown. Furthermore, it is currently unknown how much of the generalized exercise signaling network response (i.e., activation/deactivation of the breadth of kinases and substrates quantified in this study) are related to substrate utilization and disruption of energy homeostasis versus how much is mechanistically involved in the adaptive response to exercise. Functional validation experiments (e.g., target knockdown, overexpression, and/or mutagenesis) in cellular and/or animal models are required to determine their respective roles in regulating exercise-induced muscle adaptations and molecular processes, such as MTFP1’s potential functional role in regulating inner mitochondrial membrane fission/fusion in response to acute exercise.

## Conclusions

Collectively, the present study has uncovered a previously unknown breadth of shared and exercise intensity-specific molecular machinery engaged by a single bout of HIIT versus MICT in untrained healthy males. Our phosphoproteomic datasets have confirmed several canonical exercise signaling pathways known to be regulated by acute exercise independent of intensity. However, by uncovering the wider complexity of acute exercise-regulated molecular transducers including a subset of unique exercise intensity-specific phosphosites, our global approach also challenges the existing exercise physiology dogma established primarily using targeted approaches that a lack of skeletal muscle signaling differences exists in response to acute work-matched HIIT versus MICT. Exercise signaling pathways and molecular transducers within the exercise intensity-specific HIIT and MICT signaling networks warrant further functional validation and can be reinforced with longer interventions and/or repeated exercise training to potentially stimulate cardiometabolic health-promoting effects and help combat a range of chronic metabolic conditions in populations with disease.

## Supplementary Information

Below is the link to the electronic supplementary material.

Supplementary Fig. 1 Distribution of phosphosite quantification and confirmatory immunoblot analysis of canonical AMPK and ACC exercise signaling responses to HIIT and MICT (PDF 4599 kb)

Supplementary Fig. 2 Protein modules showing distinct clusters of kinase–substrate signaling profiles in response to HIIT versus MICT (PDF 3586 kb)

Supplementary Table 1 Human skeletal muscle phosphopeptides identified and quantified using TMT labeling and following Perseus data processing (XLSX 10624 kb)

Supplementary Table 2 Human skeletal muscle phosphosites identified and quantified following PhosR data normalization, with quantification rates for each phosphosite (XLSX 6229 kb)

Supplementary Table 3 Human skeletal muscle differentially regulated phosphosites from each timepoint and exercise intensity (XLSX 1769 kb)

Supplementary Table 4 Reproducibility of phosphosites at baseline between HIIT and MICT trials (XLSX 601 kb)

Supplementary Table 5 Phosphosites significantly correlated with plasma lactate concentrations from each timepoint and exercise intensity (XLSX 630 kb)

## Declarations

### Availability of data and material

This article and its Supplementary Information include all datasets generated during this study. The MS phosphoproteomics data have been deposited to the ProteomeXchange Consortium via the PRIDE [60] partner repository with the dataset identifier PXD053295 (Reviewer login details—username: reviewer_pxd053295@ebi.ac.uk; password: 1PKEot15EUSc). Further information and requests for materials and resources including raw data, code, and unique materials collected and used in this study should be directed to and will be fulfilled by the corresponding author and lead contact, Nolan J. Hoffman (nolan.hoffman@acu.edu.au).
