# Supplementary material for: Phosphoproteomics Uncovers Exercise Intensity-Specific Skeletal Muscle Signaling Networks Underlying High-Intensity Interval Training in Healthy Male Participants
Source: Sports Med. 2025 Apr 21;55(8):1983–2004. doi: 10.1007/s40279-025-02217-2 (PMC12460488; doi:10.1007/s40279-025-02217-2)

TITLE

Phosphoproteomics uncovers exercise intensity-specific skeletal muscle signaling networks underlying high-intensity interval training in healthy male participants

JOURNAL NAME

Sports Medicine

AUTHORS

Nolan J. Hoffman, Jamie Whitfield, Di Xiao, Bridget E. Radford, Veronika Suni, Ronnie Blazev, Pengyi Yang, Benjamin L. Parker, John A. Hawley

CORRESPONDING AUTHOR INFORMATION

Nolan J. Hoffman, Ph.D.

Exercise and Nutrition Research Program, Mary MacKillop Institute for Health Research

Australian Catholic University

Email: nolan.hoffman@acu.edu.au

Supplementary Figure 2

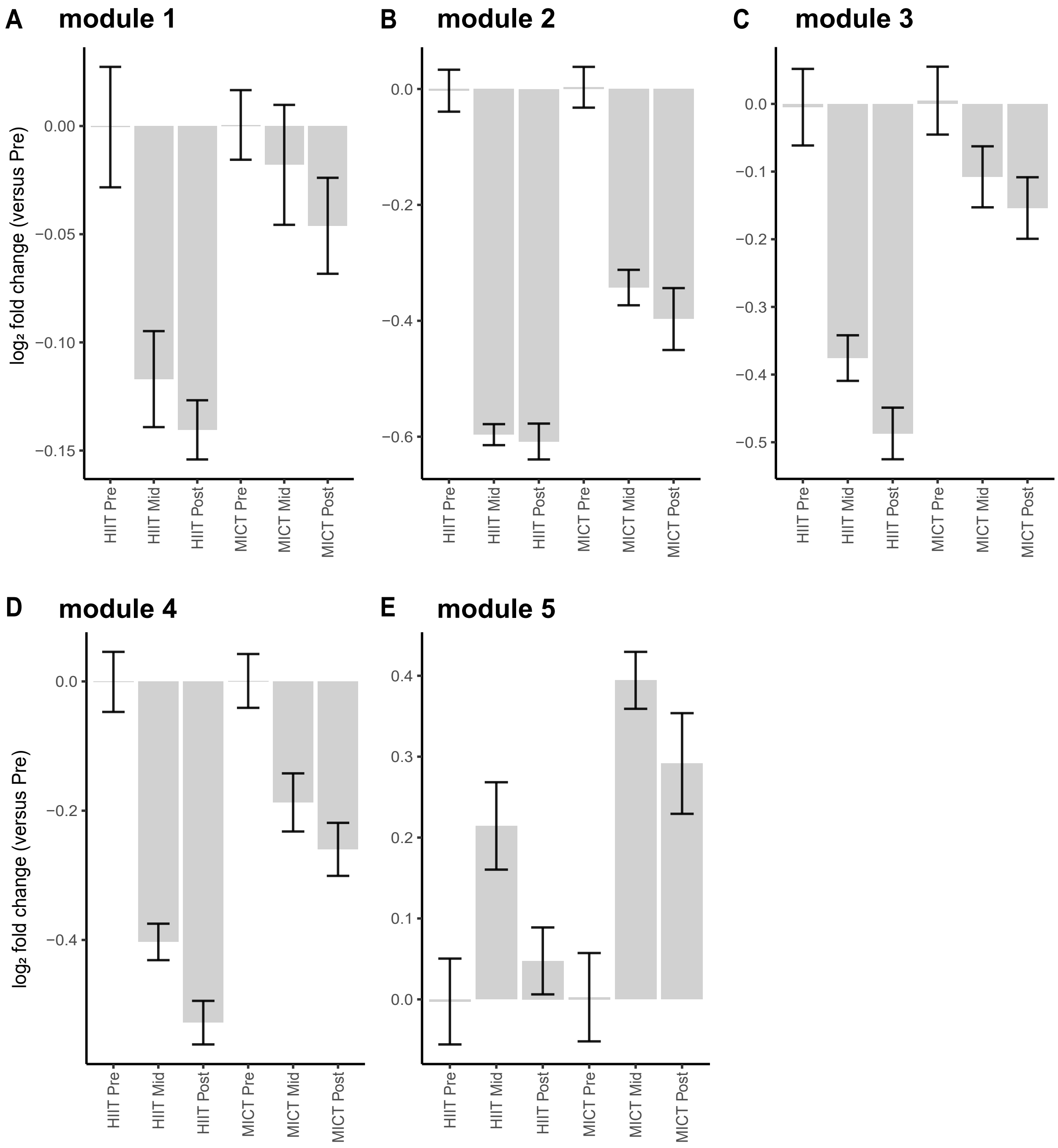

Supplement: Supplementary file 2 — Supplementary Fig. 2 Protein modules showing distinct clusters of kinase–substrate signaling profiles in response to HIIT versus MICT (PDF 3586 kb) [file 40279_2025_2217_MOESM2_ESM.pdf]
